# The Medical Service Scandal in South Africa

**Published:** 1900-08

**Authors:** 


### THE MEDICAL SERVICE SCANDAL IN SOUTH AFRICA.

BURDETT-COUTTS raised something of a breeze in England recently when he arose in Parliament and ripped into the management of the South African war in undisguised English. He had just returned from the seat of war and was full of information. His description of the suffering to which the sick and wounded were subjected, because of lack of medicines and medical attendance, sent a chill of horror over all England and do what they could the government representatives could not make a satisfactory answer. This condition is something similar to that which obtained during the Spanish-American war, although be it said in all fairness, the American army was at no time in such condition as Burdett-Coutts reports the English soldiers to have been. While it is true that there was insufficient medical attendance and too few supplies in both wars, yet the fault does not lie at the doors of the Surgeon-General of either army, nor yet are the medical departments and staffs to blame. The fault lies wholly and solely with the politicians who have not, and even yet cannot, see the necessity for a sufficiently large corps of surgeons to meet all requirements. When Surgeon-General Sternberg was brought face to face with the problems of a war he was found to be disgracefully short handed, although his corps was manned to the limit provided by the laws which a beneficent and politically infected congress had seen fit to pass. In his extremity he employed contract surgeons, as many as the law allowed, and these men without military training went in and practised medicine. Naturally, there was waste of supplies and losses of material as there was bound to be where men knew nothing of the red tape of departmental paper work; yet these men practised medicine and the world saw a well-equipped and generally competent corps of surgeons at work under Surgeon-General Sternberg in a very short time, and excellent work was done, too.

The whole fault lies with the politicians and the headquarters. The politicians cannot see the benefit of giving an army sufficient surgeons to meet emergencies which arise; they cannot see any reason why the corps should be large enough to give a certain number opportunities to study and make scientific research. They allow merely enough to do the actual work of the army. Then, too, there is the general, hidden contempt in which army medical men are held by the departments and the line; headquarters neglects them; the corps is disgracefully undermanned; surgeons are seldom mentioned in reports, except when they are killed or die from overwork. The needs of an army do not appear to be considered in connection with the medical department, the apparent and popular idea being that an army surgeon has little or nothing to do and is paid a good salary for doing it. The conditions in this country and England are largely similar at this time and the studied ignoring of the medical departments amounts to little short of absolute criminality. In South Africa the surgeons are working day and night; in the Philippines they are working night and day; nearly all the American regular army surgeons are on foreign service, only enough being retained in this country to keep department matters from hopelessly tangling. The most important, the most necessary duty the coming congress has before it is to authorise an appreciable increase in the numerical strength of the medical department.

